# Graft Selection in Contemporary Anterior Cruciate Ligament Reconstruction

**DOI:** 10.5435/JAAOSGlobal-D-21-00230

**Published:** 2021-10-25

**Authors:** Rafael A. Buerba, Stephanie A. Boden, Bryson Lesniak

**Affiliations:** From the Banner Health Medical Center, Glendale, AZ (Dr. Buerba), and UPMC Freddie Fu Sports Medicine Center, Department of Orthopaedic Surgery, University of Pittsburgh Medical Center, Pittsburgh, PA (Dr. Boden and Dr. Lesniak).

## Abstract

In the last decade, there have been significant advances in our understanding of anterior cruciate ligament (ACL) reconstruction. Recent prospective cohort studies have identified risk factors for ACL reconstruction graft failure such as younger patient age, increased activity level, nonanatomic tunnel placement, and the use of allograft. Among these, the most easily modifiable risk factor is graft choice. Given that the surgeon's recommendation has been shown to be one of the most important factors behind patients' graft choice, it is critical that the operating surgeon have a thorough understanding of all the ACL graft options available to choose the graft that would be most suitable for the patient's personalized reconstruction (i.e., patient's anatomy, sport, level of competition, age, risk factors for failure, and graft used in previous ACL surgery). The purpose of this review is to provide an up-to-date understanding of the current ACL graft reconstruction options. The indications, advantages, and disadvantages of the different ACL reconstruction graft options available will be discussed.

Injury to the anterior cruciate ligament (ACL) is one of the most common sports-related injuries, and its incidence has been increasing at all levels of competition. In the United States alone, the rates of ACL reconstruction (ACLR) have increased significantly in a 12-year period from 10.36 to 18.06 and from 22.58 to 25.42 per 100,000 person-years for females and males, respectively.^1^ Given the rise in ACL injuries and ACLR revision rates, there has been an increased interest in understanding ACLR graft choices to improve outcomes, decrease morbidity, and lower revision rates. It is thus imperative that the ACL surgeon has a complete understanding of the graft options available given that his or her recommendation will strongly influence a patient's ultimate graft choice.^2^

## Anatomy and Biomechanics of Native Anterior Cruciate Ligament

Before performing ACLR with autograft or allograft, knowledge of the anatomy and biomechanics of the native ACL is essential. The ACL functions to provide anteroposterior and rotary stability to the knee. It comprises two functional bundles based on their tibial insertion sites, the anteromedial and posterolateral (PL) bundles.^3^ These bundles work synergistically to stabilize the knee in response to anterior tibial and rotary loads.^3^ Biomechanical studies have shown that the PL bundle contributes the most to rotatory stability to the knee in lower degrees of flexion, and the anteromedial bundle provides more sagittal stability in higher degrees of flexion.^3^

On average, the midsubstance of the ACL is 10 to 11 mm wide (range 7 to 17 mm) with an average thickness of 3.9 mm and a cross-sectional area of 40.9 ± 3 mm.^2,3^ It comprises a highly organized collagen matrix with 20-μm-thick bundles of collagen fibers surrounded by loose connective tissue.^3^ Its sporadic fiber arrangement allows for a higher tensile strength than many other ligaments, with a maximum tensile strength reported as high as 2160 N (mean tensile strength approximately 1725 N), with a stiffness of 242 N/mm (mean stiffness 182 N/mm) and a strain rate of approximately 20% before failure^4^ (Table 1). Dynamic stabilizers of the knee include the surrounding musculature and aponeuroses (quadriceps femoris, extensor mechanism, pes anserinus, semimembranosus, popliteus, and biceps femoris) and are thought to rely on proprioceptive feedback regarding joint position to enhance knee stability.

**Table 1:** Biomechanical Properties of ACL Graft Options^a^

Tissue	Load to Failure (N)	Stiffness (N/mm)	Midsubstance Cross-Sectional Area (mm^2^)	Biological Incorporation
Native ACL	2160	242	40.9 ± 3	—
Autograft				
Quadruple HS	4100	776	55.3 ± 8.0	Graft-to-bone healing (8-12 wk)
BPTB^b^	2977	620	33.2 ± 7.3	Bone-to-bone healing (6 wk)
QT^b^	2352	463	71.4 ± 10.5	Bone-to-bone and graft-to-bone healing (6-12 wk)
Allograft				
BPTB	1403	224	—	Bone-to-bone healing, slow incorporation (>6 mo)
Achilles tendon	1189	741	105	Bone-to-bone and graft-to-bone healing, slow incorporation (>6 mo)
Tibialis anterior	3012	343	—	Graft-to-bone healing, slow incorporation (>6 mo)

ACL = anterior cruciate ligament, BPTB = bone–patellar tendon–bone, HS, hamstring, QT = quadriceps tendon

a Adopted from data by West and Harner^4^, Shani et al^5^, and Lin et al.^6^

b There are discrepancies in the literature regarding ultimate load to failure and stiffness for BPTB autograft and QT autograft. Those values reported on the table are adopted from West and Harner^4^ and indicate that BPTB has a greater load to failure and stiffness than QT. A more recent study by Shani et al.^5^ showed the opposite. In this study, the QT mean load to failure and stiffness were 2185.9 ± 758.8 N and 466.2 ± 133 N/mm, respectively, and the BPTB mean load to failure and stiffness were 1580.6 ± 479.4 N and 278.0 ± 75 N/mm, respectively.

## Graft Choices

There are many graft choices for ACLR, including autografts and allografts. The autograft choices most commonly used include hamstring (HS), bone–patellar tendon–bone (BPTB), and quadriceps tendon (QT) with or without a bone block.^7^ Iliotibial band autografts have also been described for use in both primary ACLR and as an augmentation technique, largely in the skeletally immature patient population.^8^ For allografts, the most common choices include BPTB, tibialis anterior or posterior, HS, and Achilles tendon.^7^ Iliotibial band and peroneal allografts have also been described for ACLR.^9^

The ideal graft for use in ACLR should have similar properties to those of the native ligament, limit donor site morbidity, and allow for secure fixation and rapid incorporation.^4^ The choice of graft should also be individualized to the patient's risk factors for failure, anatomy, sport, level of competition, age, and graft used in previous ACL surgery for cases of revision reconstruction.^4,7,10^ Graft size is an important consideration, as larger grafts may provide improved strength, but have also been shown to increase the risk of postoperative arthrofibrosis.^11^ Overall, graft size, rather than graft type, seems to be more associated with the risk of arthrofibrosis after ACLR.^11^ Recent prospective cohort studies have identified several risk factors for ACLR graft failure.^7^

Younger patient age has been consistently shown to portend a higher risk of reconstruction failure, which is likely in part related to increased activity level and demands on the reconstructed graft.^7^ Higher activity level at the time of ligament injury has been associated with an increased risk of graft rupture.^7^ Studies have also clearly demonstrated that tunnel malposition is associated with higher rates of reconstruction failure.^7,12^ The use of allograft in comparison to autograft has also been identified as a risk factor for reconstruction failure, especially in younger more active patients.^7^ It is important to note that regardless of graft type, anatomic ACLR is considered the benchmark, reconstructive technique,^13^ as it has been shown that anatomic ACLRs reduce the risk of posttraumatic osteoarthritis (OA) at long-term follow-up.^14^ Given the multitude of graft options and risk factors for failure, it is critical that the operating surgeon have a comprehensive discussion with the patient regarding which graft option(s) would be most suitable for the patient's individualized reconstruction, particularly given that the surgeon's recommendation has been shown to be one of the most important factors behind the patient's graft choice.^2^ A summary of the indications, advantages, and disadvantages of the different ACLR graft options is shown in Table 1.

## Autograft

Autografts remain the preferred graft choice in the young athletic patient population due to higher rates of failure, increased costs, and risk of repeat rupture associated with ACL allograft reconstruction.^4^ Autograft options include HS, BPTB, and QT with or without a bone block.^4,10^ Autografts can be harvested from the ipsilateral/injured side or from the contralateral or uninjured extremity. There are advantages and disadvantages to the different types of autografts, which will be discussed in detail. As previously mentioned, it is imperative to tailor graft choice to the patient's anatomy, sport, age, level of competition, and graft used in previous ACL surgery.^4,7,10^

## Bone–Patellar Tendon–Bone Autograft

**Figure 1:**
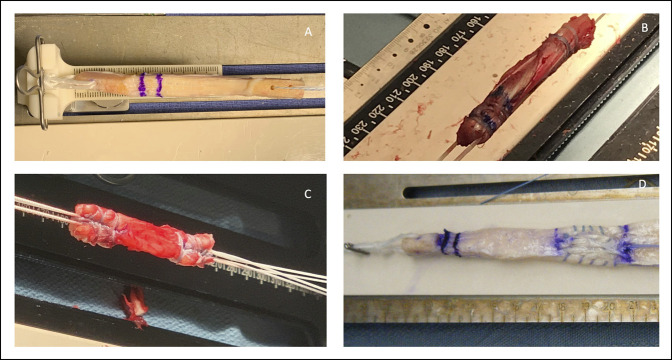
Intraoperative images of (**A**) BPTB autograft, (**B**) quadrupled HS tendon autograft, (**C**) soft-tissue QT tendon autograft, and (**D**) Achilles tendon with bone block allograft after preparation. This graft is prepped long so that it can be used as an LET if need be. BPTB = bone–patellar tendon–bone, HS = hamstring, LET = lateral extra-articular tenodesis, QT = quadriceps tendon

The BPTB autograft consists of a portion of the central aspect of the patellar tendon with its corresponding bone plugs from the patella and tibia (Figure 1, A). The BPTB autograft has been historically considered the benchmark graft for ACLR, mostly because of its long-standing track record and widespread use.^7^

Past randomized controlled trials of primary ACLRs comparing HS versus. BPTB autografts have shown similar results, patient-reported outcomes, and incidences of postoperative OA on follow-up radiographs.^15^ Other long-term outcome studies have demonstrated a higher incidence of post-traumatic OA after ACLR when using BPTB autograft in comparison with HS autografts. The rates of OA have been reported to be as high as 39% at 10 years after BPTB compared to 18% at 10 years after HS autograft and the relative risk for OA for BPTB versus HS has been reported to be 1.61.^16,17^ More recent studies and meta-analyses have found that BPTB autografts have lower failure rates and higher return-to-sport rates compared with HS autografts, especially in the young athletic patient population.^18-20^ These studies have reported graft rupture rates between 1.9% and 6.6% after BTB autografts compared with 4.9% and 17.5% after HS autografts. Some studies have also found less residual anterior knee laxity and improved stability with the use of BPTB autograft versus HS autograft at longer-term follow-up.^16,19^ Proponents of BPTB also cite the advantages of bone-to-bone healing compared with soft-tissue allografts. In a sense, bone-to-bone healing is similar to fracture healing and is faster and stronger than soft-tissue healing.^4^ Although bone grafts have been shown to heal to host bone within 6 weeks, soft-tissue grafts take 8 to 12 weeks to fully incorporate.^4^

Although outcomes with BPTB autograft are consistently good, there are complications associated with BPTB that are almost exclusively related to the graft harvesting technique. These harvest-related complications include patellar fractures (0% to 2%), patellar tendon rupture (0.25%), and patellar tendonitis as shown in Table 2.^4^ It should be noted that intraoperative and postoperative patellar fractures and patellar tendon ruptures are uncommon complications after BTB harvest.^4,6^ One of the most common complications after ACLR that has been associated with BPTB autograft use is anterior knee pain.^16,19^ In a meta-analysis of 21 studies, BPTB autografts had an incidence of anterior knee pain of 17.4% versus 11.5% in HS autografts.^19^ The cause of postoperative anterior knee pain has not been well elucidated, but it has been hypothesized that anterior knee pain is related to the harvesting method of the BPTB graft. Studies have shown that patients who kneel for a profession or sport (eg, wrestlers, plumbers, and mechanics) experience more postoperative anterior knee complications—making BPTB autograft a relatively contraindicated graft choice in these patient populations.^16^ There is some evidence that anterior knee pain after ACL surgery may be more related to loss of motion and poor rehabilitation rather than graft choice, and studies have demonstrated a decrease in anterior knee symptoms after initiation of an accelerated rehabilitation program that emphasizes knee extension.^4^ BPTB autograft is also contraindicated in skeletally immature individuals as the graft harvest and fixation methods would violate the physes and increase the risk of growth arrest.^16^

**Table 2:** Summary of Indications, Advantages, and Disadvantages of Different ACL Reconstruction Graft Options

Graft Type	Indications	Advantages	Disadvantages	Complications
Allograft	•Patients aged >40 years •Multiligament knee injuries •Patient preference •Previous harvest from other donor sites •Inadequate autograft tissue	•Decreased surgical time •Predictable graft size •Decreased morbidity •Easier recovery •Double-bundle reconstructions •Over-the-top reconstructions •Multiple types of allograft available (eg, patellar tendon, tibialis anterior or posterior, HSs, peroneals, iliotibial band, and Achilles tendon)	•Cost •Infectious disease transmission •Delayed incorporation •Higher failure rate (up to 25%)^21^ •Lower return-to-sport rate compared with autograft (43.3% versus 75%)^22^	•Infection •Intraoperative fracture of allograft bone given its softness
Autograft				
BPTB	•Young, athletic individuals who are skeletally mature^16^ •Sports or professions that do not involve kneeling^8^	•Reliable, time-tested results^7^ •Fastest incorporation and healing (6 weeks versus 8-12 weeks)^4^ •Good outcomes in young, active patients^7,15^ •Improved rates of return to sport compared with HS^4,18-20^ •Lower revision rate compared with HS (1.9%-6.6% versus 4.9%-17.5%)^18-20^	•Donor site morbidity (anterior knee pain 17.4%)^19^ •Risk of patellar fracture •Contraindicated in skeletally immature patients •Increased risk of OA compared with HS (39% versus 18% at 10 years)^16,17^	•Patellar fracture (zero-2%)^4^ •Patellar tendon rupture (0.25%)^4^ •Patellar tendonitis •Anterior knee pain (17.4%)^4,19^
HS	•Young, athletic individuals who are skeletally mature or immature •Sports that do not rely heavily on HSs (ie, sprinters)	•Option in skeletally immature patients •Greater cross-sectional area than BTB (53 versus 35 mm^2^)^3,16^ •Maintenance of the integrity of the extensor mechanism^16^ •Lower harvest site morbidity •Smaller incision than BTB^4^ •Less postoperative knee pain than BTB (11.5% versus 17.4%)^19^	•Donor site morbidity (knee flexion weakness)^16^ •Less predictable graft size^16^ •Compromise of medial knee structures^16^ •Higher rerupture rates than other autografts (17.5% versus 6.4% BTB)^20^ •Longer graft integration times (8-12 weeks)^4^ •Higher infection rates (0.6% versus 0.07% BTB)^23^	•Bone tunnel enlargement due to windshield wiper effect^24^ •Residual HS weakness^16^
QT	•Young, athletic individuals who are skeletally mature or immature (no bone block) •ACL with large footprint (>16 mm) •Athletes who rely on their HSs (sprinters) •Athletes or laborers who spend time on their knees (eg, wrestlers, judo, and carpenters)	•Reliable and robust graft (cross-sectional area 62 mm^2^)^4^ •Can be used with or without bone block •Less risk of infection compared with HS •Less risk of injury to the infrasaphenous branch (1.5% versus 53.3% in BTB)^13^ •Low donor site morbidity (zero-15% versus 18%-51% in BTB)^13,17,25^ •Less anterior knee pain (4.6% versus 26.7% in BTB)^13^ •Can be harvested minimally invasively	•Prolonged quadriceps weakness with full-thickness grafts •Donor site pain •Fluid extravasation during arthroscopy	•Postoperative hematoma^25^ •Patellar fracture with bone block harvest^25^ •Rectus femoris retraction^25^

ACL = anterior cruciate ligament, HS = hamstring, QT = quadriceps tendon

## Hamstring Autograft

Given the complications and disadvantages associated with BPTB autografts, HS autografts (semitendinosus and/or gracilis muscle tendons) are a viable option for ACLR in certain patient populations.^16^ HS autografts are typically folded on themselves to increase graft diameter and strength and can be folded to form two- to six-strand grafts.^3^ Although larger grafts are biomechanically stronger, grafts that are too large can cause impingement. Quadrupled HS grafts are most commonly used as they provide a balance between strength, stiffness, and mobility.^3^ A quadrupled HS autograft after harvest and preparation is shown in Figure 1, B. The advantages of HS autograft reconstruction are shown in Table 2. These include greater cross-sectional area, avoidance of the extensor mechanism in the graft harvesting process, and that it is an option for ACLR in the skeletally immature.^16^ Disadvantages include prolonged healing times, unpredictable graft size, higher failure rates in certain patient populations, and knee flexion weakness.^16^ HS autografts have been known to cause bone tunnel enlargement due to a windshield wiper effect from the suspensory fixation.^24^ There is also a risk of residual HS weakness,^16^ making the graft a relative contraindication for athletes who heavily rely on their HSs for their athletic performance (ie, sprinters). HS autografts have also been shown to have higher infection rates than BPTB autografts; a registry study from Kaiser Permanente demonstrated that patients who had HS autograft ACLR were 8.24 times more likely to develop a deep surgical site infection than patients who had a BTB autograft (0.6% compared with 0.07%).^23^

The main concern after HS autograft ACLR is graft failure. As previously discussed, recent studies and meta-analyses have found that HS autografts have higher failure rates compared with BPTB autografts.^18^ In particular, HS autograft seems to fail more among younger female patients, with graft rupture reported at 17.5% after HS autograft compared with 6.4% after BTB autograft in females aged 15 to 20 years.^20^ Interestingly, a recent randomized controlled trial showed that the addition of a lateral extra-articular tenodesis (LET) to HS autograft ACLRs resulted in a clinically relevant and statistically significant reduction in graft failure and persistent rotatory laxity at two years after surgery.^26^ Despite the improvements in graft rupture rates and postoperative laxity, there were no clinically important differences noted in patient-reported outcome meausures in this study. This is a promising result, and further research is currently being performed with regard to indications and long-term outcomes after LET.

## Quadriceps Tendon Autograft

The QT autograft is an option for ACLR in the primary and revision setting and has recently gained popularity due to its versatility and more recent outcome data (Figure 1, C). The QT can be harvested as a full-thickness (FT) or partial-thickness graft with or without a bone block.^13^ Harvesting with a bone block can provide a longer graft if needed. The QT is a reliable and robust graft with a cross-sectional area up to twice that of a BPTB autograft.^5^ The graft is longer and wider, has a higher tensile strength, about 50% more mass than a BPTB autograft, and has been shown to be biomechanically similar to the six-strand HS autograft with regard to ultimate load to failure.^10^ It can be used in both primary and revision ACLRs based on tunnel size, tunnel position, previous graft used, etc.^13^ QT harvest is associated with less damage at the tendon harvest site than BPTB harvest, with similar patient-reported outcomes.^10^ In addition, it can be harvested with a minimally invasive technique using a small incision with or without the assistance of an arthroscope.^27^

Compared with other autograft options, the QT has been shown to have lower risks of anterior knee pain (4.6% versus 26.7% in BTB), less injury to the infrasaphenous nerve branch (1.5% versus 53.3% BTB), less donor site morbidity (zero to 15% versus 18% to 51% BTB), and a low rate of quad strength deficit for both FT and partial-thickness (PT) grafts (89.5% FT and 85.1% PT as a percentage of the contralateral side).^13^ The all soft-tissue QT has become increasingly used in skeletally immature patients and is a good alternative to the traditional HS autograft with improved outcomes, as previously discussed. Despite its advantages, QT autograft ACLR is not without its associated complications (Table 1). These include donor site pain (although less common than BPTB–zero to 15% after QT versus 18% to 51% after BTB), bleeding (if the quadriceps muscle is violated), retraction of the rectus femoris (occurs rarely and correlates with graft harvest that spans the myotendinous junction), and fractures of the patella after QT harvest with bone block.^25^

Whereas initial outcome data were unfavorable for QT autografts, more recent data using newer harvest techniques have provided support for the use of QT autografts in both the primary and revision settings.^5,27^ In a systematic review of clinical studies (Level of Evidence III, systematic review of Level I, II, and III studies) that evaluated QT versus BPTB and HS, no significant differences were found in laxity, patient-reported outcomes, or patient satisfaction between QT and either BPTB or HS.^25^ In addition, a more recent meta-analysis evaluating 27 clinical studies with 2856 patients (Level of Evidence II) concluded that QT had similar graft survival rates and comparable functional and clinical outcomes when compared with BPTB and HS autografts.^28^ In this meta-analysis QT also showed improved functional outcomes compared with HS autograft and significantly less harvest site pain compared with BPTB autograft. Numerous studies have also shown no significant differences in knee stability testing (ie, KT-1000, pivot shift rating, and Lachman testing) between QT versus HS or BPTB.^25^ A recent registry study did show a higher revision rate for QT (4.7%) versus HS and BPTB (2.3% versus 1.5%. respectively), although QT patients in this study comprised only 3.2% of the patient sample and graft size, fixation technique, and bone block use were not available for analysis.^29^ Because the QT is historically the least used autograft option for ACLR, further research is required to better understand its long-term outcomes.^7^

## Allograft

As discussed previously, a variety of fresh-frozen allograft options exist such as patellar tendon, tibialis anterior or posterior tendons, HS tendons, peroneal tendons, iliotibial band, and Achilles tendon.^9^ An example of a prepared Achilles tendon allograft for ACLR is shown in Figure 1, D. This graft is prepped long so that it can be used for LET if needed. The main advantages of allografts over autografts are shorter surgical times, predictable graft size, no harvest site morbidity, and easier recovery in the immediate postoperative period.^30^ Disadvantages of allografts include concerns for disease transmission, immune responses, weakening of graft tissues that occurs due to sterilization and processing techniques, and delayed incorporation and healing.^7,31^ Other disadvantages are that allografts are more costly than autografts^4^ and that they are not as widely available in other countries outside of the United States.^30^

Before proceeding with allograft ACLR, it is imperative that surgeons be familiar with graft processing techniques and infection risk, as different techniques have been shown to affect risks of graft failure and subsequent revision surgery.^32^ Allograft processing begins with careful screening of the donor patient to minimize the risk of communicable diseases.^31^ After allograft harvest, grafts must be sterilized before they can be stored for eventual transport and usage for ACLR. There are a number of sterilization methods including ethylene oxide, low- or high-dose gamma irradiation, and supercritical carbon monoxide among other techniques. All methods of allograft sterilization have been found to compromise the structural integrity of the allograft to varying degrees.^6,21^ Gamma irradiation has been found to have a dose-dependent relationship with delayed graft healing and increased risk of failure.^21,32^ Newer sterilization techniques including supercritical carbon dioxide and the use of gamma irradiation in conjunction with antioxidants have been developed and have yet to demonstrate significant improvement in mechanical properties of the grafts in comparison to previous methods.^21,32^ With graft processing and sterilization, risk of disease transmission with allografts is extremely small, and risk of surgical infection has not been shown to differ markedly in comparison with autografts.^21,23,31^ Allografts can be stored as fresh frozen, freeze dried, or cryopreserved and typically remain frozen for approximately 2 to 4 weeks until serologic studies are complete to confirm that the grafts are disease free.

Regarding outcomes after ACLR with allograft, studies have consistently shown that allografts have a higher rerupture rate than autografts in young, athletic individuals.^22,33^ A recent systematic review demonstrated that in active patients aged <25 years, there was a 9.6% graft rupture rate with autograft versus 25.0% with allograft.^33^ There is also evidence that athletes have an improved return-to-sport rate when undergoing ACLR with autograft compared with allograft, with one study demonstrating a return-to-sport rate of 43.3% after BTPB allograft compared with 75.0% after BTPB autograft.^34^

Although the literature demonstrates higher failure rates with allograft in young, active athletes, there is a compelling role for allograft use in the older, less active patient population.^7^ In addition to decreased morbidity, easier recovery, and shorter surgical times,^30^ studies have demonstrated that outcomes and revision rates after allograft use in patients who are aged >40 years are consistently similar to those after autograft ACLRs.^35^ There are special circumstances in which allografts can be useful, particularly in younger patients. These include multiligament knee injuries, multiple-revised ACLRs with limited autograft options, patient preference, and cases in which autograft tissue is inadequate.^31^ A summary of relative indications for allograft ACLR is shown in Table 2.

## Biomechanics of Anterior Cruciate Ligament Grafts

The biomechanical profiles of the native ACL, as well as autografts and allografts used for ACLR, are shown in Table 1. Although the biomechanical properties of various grafts have been evaluated extensively in the literature,^4,19,23^ it is important to note that comparison of absolute values among these studies is difficult due to differences in donor age, graft size, and testing methodology.

HS autografts usually consist of semitendinosus and gracilis tendons folded on themselves to make a four-strand graft to increase the graft diameter and strength. Numerous biomechanical studies have demonstrated that the four-strand HS autograft provides the greatest tensile strength and stiffness in comparison to the BPTB, QT, and allograft options^3,5^ (Table 1). The ultimate failure load of this quadrupled HS autograft has been shown to occur around 4100 N with a stiffness around 776 N/mm.^3,4^ Despite its superior strength in biomechanical studies, the HS autograft has been shown to have variable healing as it relies on soft tissue–to–bone healing, which likely translates to diminished mechanical properties after transplantation.^4^

BPTB autografts consist of patellar tendon attached to bone plugs proximally and distally, allowing a bone-to-bone interface for healing. Biomechanical studies of the BPTB autograft have demonstrated that its overall strength is related to its cross-sectional area.^3^ The tensile load of the BPTB autograft has been shown to be approximately 2900 N with a stiffness around 270 N/mm.^3,4^ More recent studies have shown these values to be less than originally reported with BPTB autograft, having a mean load to failure of 1580.6 ± 479.4 N and a stiffness of 278.0 ± 75 N/mm.^5^ Although the overall strength and stiffness of the BPTB autograft is less than that of the HS autograft, the BPTB autograft is still stronger than the native ACL; furthermore, the bone-to-bone interface of the BPTB portends to improved healing.^3,4^

The QT autograft is a trilaminar graft comprised of tendons from the rectus femoris, vastus medialis, and vastus lateralis.^13^ Although initial studies demonstrated inferior biomechanical properties for the quadriceps tendon autograft, these studies used a QT substitution graft that consisted of the QT–prepatellar retinaculum–partial-thickness construct that is vastly biomechanically different from the modern QT construct.^27^ Newer graft harvest techniques have demonstrated improved characteristics, making it a promising alternative graft option.^5,27^ As previously mentioned, the QT autograft can be harvested as a FT graft or a partial-thickness graft with or without a bone block.^13^ Shani et al^5^ demonstrated ultimate load to failure of 2185.9 N compared with 1580.5 N with the BPTB with stiffness of 466 N/mm and 278 N/mm, respectively. The mean cross-sectional areas of the QT autograft has also been shown to be reliably larger than that of the patellar tendon autograft^5,13^ (Table 1).

Allografts overall demonstrate comparable strength and stiffness in comparison to autografts at the time of ACLR; however, as previously discussed, they have been found to have variable mechanical strength over time.^31^ Because of limitations in testing methodology, it is difficult to directly compare strength of autograft and allograft ACLR; however, strength testing in canine ACLR has demonstrated inferior maximum tensile strength of allografts in comparison to autografts, but strength is greater than that of the native ACL^6^ (Table 1). Tensile strength and modulus of elasticity of allografts have also been shown to negatively correlate with increasing donor age.^6^ Allografts undergo a process of incorporation after implantation similar to that of autografts, which includes graft necrosis, cellular repopulation, revascularization, and remodeling; however, allografts demonstrate a slower time to healing and graft incorporation.^4^

## Graft Fixation Methods

There are many graft fixation methods for ACLR and a thorough discussion of each would go beyond the scope of this review. In general, fixation methods include interference screws (aperture fixation), suspensory fixation with fixed or adjustable loops, buttons, or a combination of aperture fixation for one side of the graft and suspensory fixation for the other. Both fixation types can be used for soft-tissue grafts and grafts with bone blocks.

Historically, interference screws were metallic, most often titanium. Titanium interference screws in ACLR have been associated with a low complication profile and excellent long-term results. Despite their initial widespread use, metallic interference screws have unique disadvantages including the possibility of iatrogenic graft damage with screw insertion, limitations in postoperative advanced imaging related to metal artifact, and retention causing potential difficulties in the event of revision surgery.^36^ In light of these issues, nonmetallic interference screws have been developed as an alternative fixation method.

Nonmetallic interference screws use a variety of materials including polyglycolic acid, poly-L-lactic acid (PLLA), poly-D,L-lactic acid, poly-D,L-lactic acid with trimethylene carbonate, polyglycolic acid with trimethylene carbonate, and PLLA with β-tricalcium phosphate.^36^ Despite the initial appeal, these interference screws have raised concerns due to reports of screw breakage, increased postoperative effusion, and diminished pullout strength in comparison to metallic screws. A recent prospective, randomized controlled trial evaluated long-term outcomes of ACLR using either PLLA-HA screws versus titanium screws and found equivalent clinical results lasting up to 13 years postoperatively.^36^

Given that interference screws have the potential for graft damage and for hindering graft-to-bone healing, a variety of suspensory fixation devices have been developed, including fixed and adjustable suspensory devices.^37^ Suspensory fixation is also advantageous in pediatric patients to avoid the potential damage to the physis that an interference screw placed across the physis can cause. Concerns with suspensory fixation devices are that when grafts are placed nonanatomically, there can be bone tunnel enlargement due to a windshield wiper motion of the graft.^24^ A recent prospective study demonstrated that both fixed loop and adjustable loop suspensory devices are equally effective fixation methods for ACLR.^37^

## Summary

The ideal graft choice for ACLR depends on many patient factors and should be individualized to best match the patient's anatomy, age, needs, and expectations. A number of autograft and allograft options are available that can achieve satisfactory results if applied appropriately. The treating surgeon should thus be familiar with all the ACLR reconstruction options available to individualize and optimize each patient's treatment and outcomes.
